# Supplementary material for: Genomic analysis of shiga toxin-containing Escherichia coli O157:H7 isolated from Argentinean cattle
Source: PLoS One. 2021 Oct 28;16(10):e0258753. doi: 10.1371/journal.pone.0258753 (PMC8553066; doi:10.1371/journal.pone.0258753)

A Evidence of 7.1Anguil inversion:

- Read mapping. There was no evidence against the inversion. Long read spans the inversion points.
- Long PCR: primers were designed to span the inversion points, outside the rRNA operon. Amplified fragments were ~6kb.

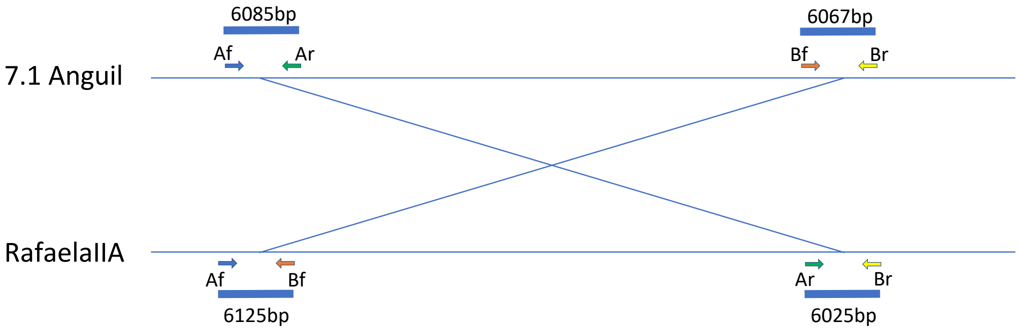

B *In silico* PCR predictions based on the assembled genomes, and using Rafaela II as a "control" with the 4 possible amplicon combinations:

Strain	Af-Ar (1)	Bf-Br (2)	Af-Bf (3)	Ar-Br (4)
**7.1Anguil**	6085bp	6067bp	--	--
**RafaelaIIA**	--	--	6125bp	6025bp

7.1 should amplify (1) and (2), whicle Rafaela II should amplify (3) and (4)

C Agarose gel of amplified fragments. RafaellaII showed expected amplicons (3) and (4), but not (1) or (2). 7.1Anguil amplified as expected the amplicon (1) but amplicon (2) showed some trouble. All reactions showed slightly amplification of unespecif fragments (equal between isolates), but correct amplicons are cleary distinguishable.

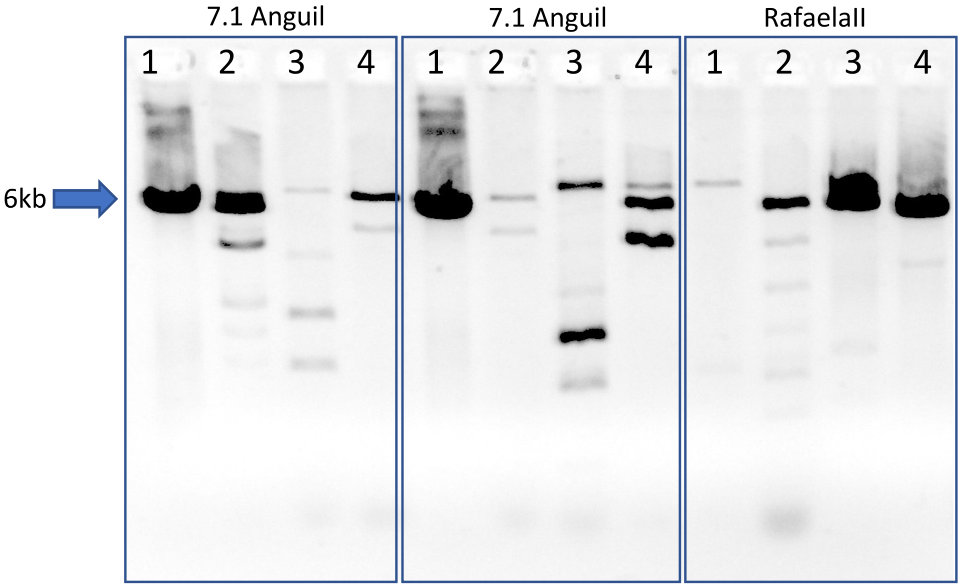

Supplement: S1 Fig — A) Evidence and graphic representation primers for combinatorial PCR to evaluate inversion. B) In silico prediction of amplicons using assembled genomes. C) Agarose gel of amplified fragments. All primers pairs were tested on 2 isolates from 7.1Anguil and one from RafaelaIIA. (PDF) [file pone.0258753.s001.pdf]
