# Supplementary figures and images for: Genomic analysis of shiga toxin-containing Escherichia coli O157:H7 isolated from Argentinean cattle
Source: PLoS One. 2021 Oct 28;16(10):e0258753. doi: 10.1371/journal.pone.0258753 (PMC8553066; doi:10.1371/journal.pone.0258753)

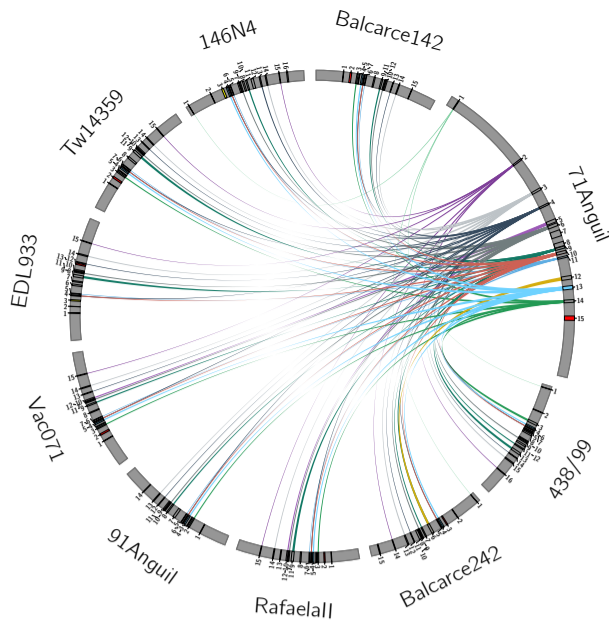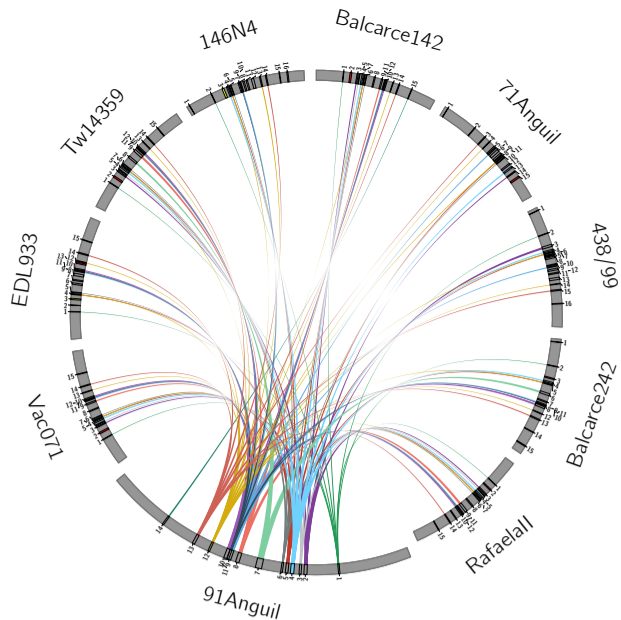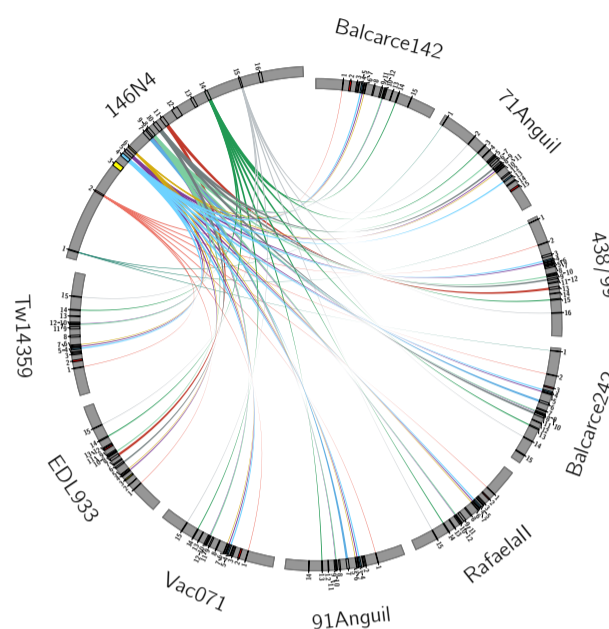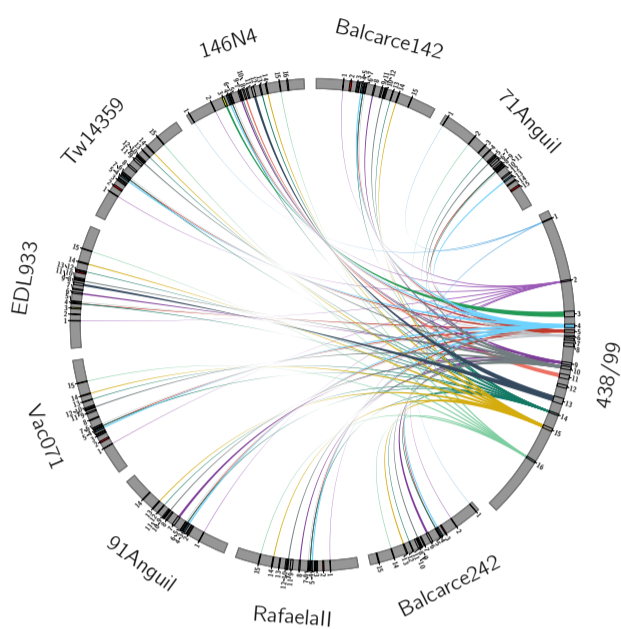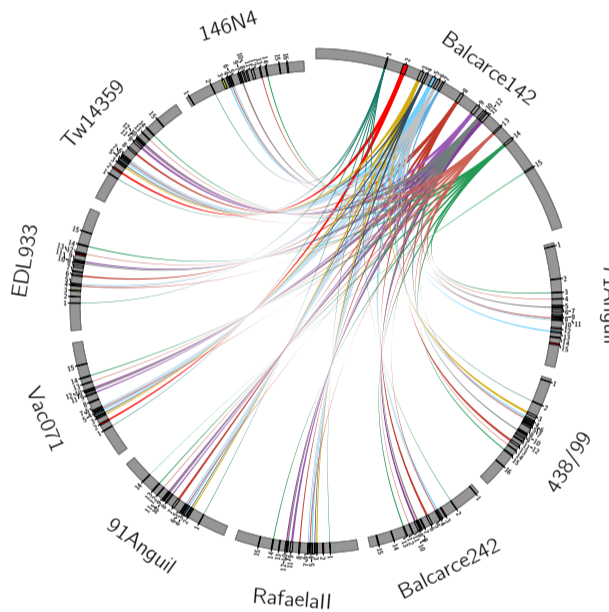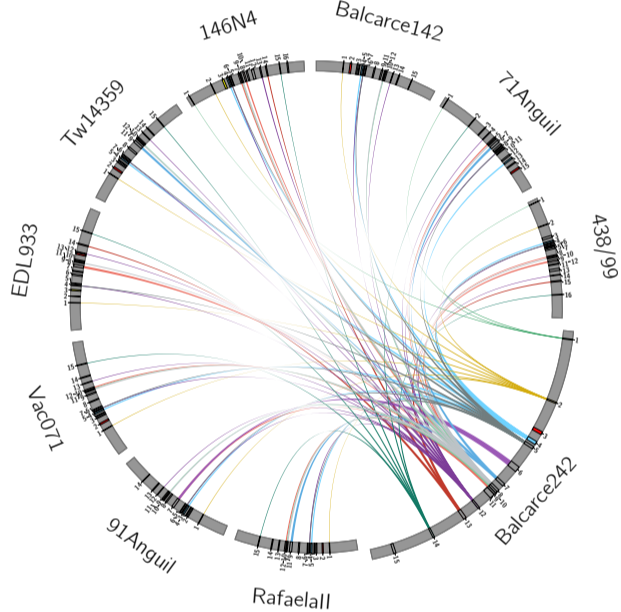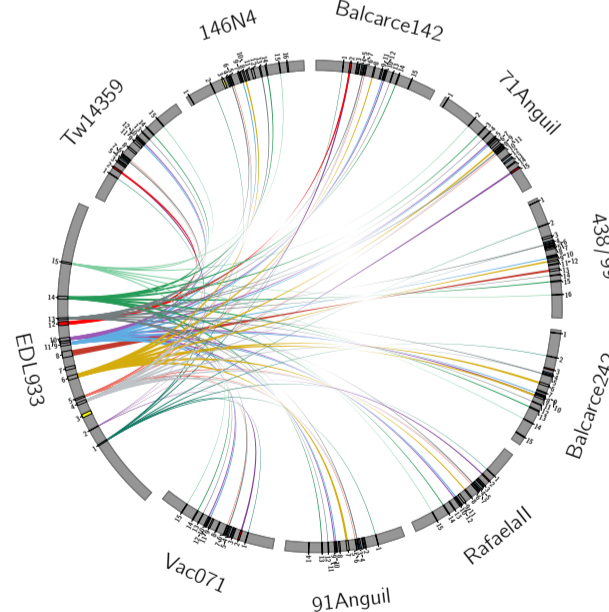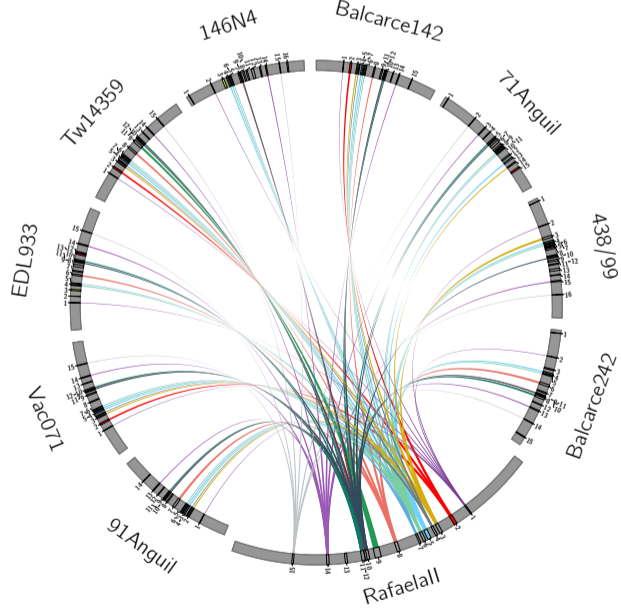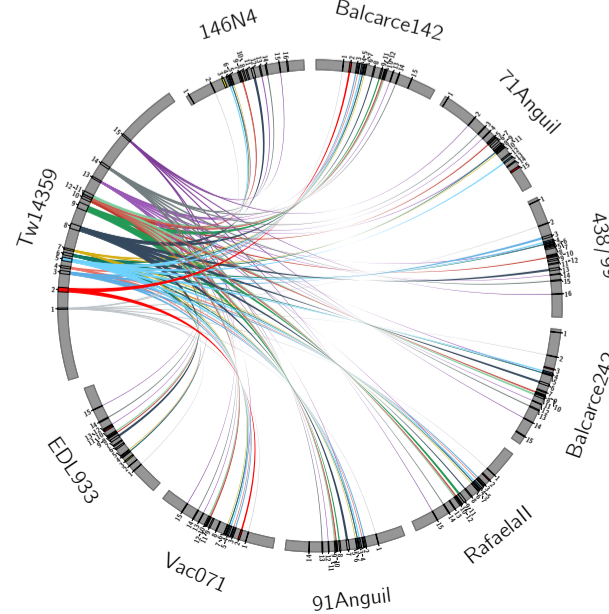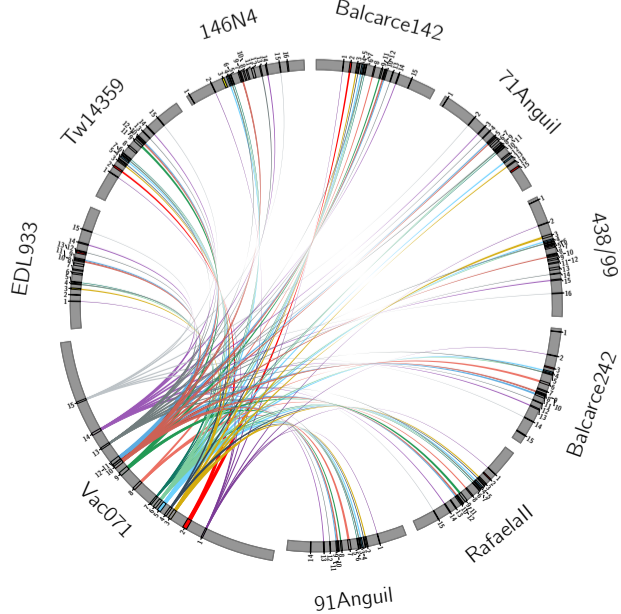

Supplement: S2 Fig — Prophage similarity among genomes of the analyzed strains. Each prophage is represented by a different color on each chromosome. On each circle, genome used as query is showed larger than the subject genomes. Lines starts on query prophages and end on every subject prophage hit showing at least 80% coverage. Size of prophages are represented in scale. For this representation Circos was used. (PDF) [file pone.0258753.s002.pdf]

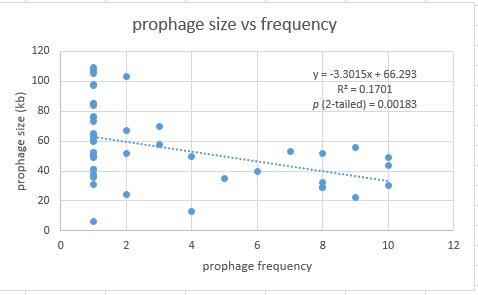

Supplement: S3 Fig — Sizes of prophage families are represented in a scatter graph against the frequency of finding a given prophage family in the 10 genome studied. R2 parameter and the tendency formula were calculated using excel. To determine the relationship between two variables Rho Spearman calculator was used (https://www.socscistatistics.com/tests/spearman/default2.aspx). rho was -0.4341. Shorter prophages were associated with higher clustering (p<0.01). (JPG) [file pone.0258753.s003.JPG]
