# Supplementary material for: Genomic analysis of shiga toxin-containing Escherichia coli O157:H7 isolated from Argentinean cattle
Source: PLoS One. 2021 Oct 28;16(10):e0258753. doi: 10.1371/journal.pone.0258753 (PMC8553066; doi:10.1371/journal.pone.0258753)
Supplement: S3 Table — (DOCX) [file pone.0258753.s006.docx]

| Family | Number of prophages | Size range (kb) | Virulence and effector genes | Relevant gene in the vicinity of prophage |
| --- | --- | --- | --- | --- |
| A | 9 | 19-23 | T3SS effectors espW, espM2 |  |
| C | 8 | 50-52 | Not identified | two component sensor-kinase and a ABC transporter ATP binding protein |
| E | 9 | 55-61 | OspB like T3SS effector | none |
| F | 10 | 44 | effector tccp and espJ | none |
| G | 10 | 27-28 | Not identified | enzyme responsible for phosphatidylglycerol and cardiolipin biosynthesis |
| J | 10 | 49 | Not identified | none |
| K | 8 | 29-34 | espK and espQ T3SS effectors | none |
| L | 7 | 51-53 | Not identified | EspX7 |
| M | 8 | 27-30 | Not identified | none |
| N | 10 | 39-41 | Not identified | effectors nleC, nleB1, nleD and nleH1 |
| O | 8 | 26-29 | Not identified | none |
