# Supplementary material for: Genomic analysis of shiga toxin-containing Escherichia coli O157:H7 isolated from Argentinean cattle
Source: PLoS One. 2021 Oct 28;16(10):e0258753. doi: 10.1371/journal.pone.0258753 (PMC8553066; doi:10.1371/journal.pone.0258753)
Supplement: Attachment — Submitted filename: Rebuttal letter.docx [file pone.0258753.s008.docx]

Mark Eppinger
Academic Editor
PLOS ONE

21 August 2021

Ref: PONE-D-21-18023
Genomic analysis of shiga toxin-containing Escherichia coli O157:H7 isolated from Argentinean cattle

Please find attached a revised version of our manuscript: “Genomic analysis of shiga toxin-containing Escherichia coli O157:H7 isolated from Argentinean cattle”. We thank the reviewers for their comments and suggestions and have modified the manuscript accordingly. Please find below a point-by-point response to each of the concerns raised by the reviewers.

We feel that these changes have significantly enhanced our manuscript, and hope that you will now find it acceptable for publication in *Plos One*.

Yours sincerely

Dr. Angel Cataldi

**Responses to the academic editor:**

Comment/Question (C/Q): Quality of the figures was borderline when sent out for initial review and I would appreciate if you could include higher quality figures and label them accordingly.

Answer (A): We apologize for the low quality of the figures when the pdf to submit was ready. Now we have uploaded high definition tiff figures.

C/Q: Please also address the concerns raised by reviewer 2 in regards to data/software availability.

A: all genome data is now available.

C/Q Considering the limited number of isolates analyzed conclusions should not be overstated. As suggested by reviewer 2 "Results/Discussion" could be shortened to avoid redundancies without hurting the overall information content of this manuscript.

A: As suggested Results and Discussion were combined in a single section. This combination removed much redundancy. Part of the text from the discussion of the original manuscript submitted is distributed in lines 204-207, 233-236, 240-249, 312-317, 320-327, 356-360, 418-432, 444-447, 459-466, and 501-512 of the revised clean manuscript.

**Journal Requirements:**

1. Please ensure that your manuscript meets PLOS ONE's style requirements, including those for file naming. The PLOS ONE style templates can be found at 
<https://journals.plos.org/plosone/s/file?id=wjVg/PLOSOne_formatting_sample_main_body.pdf> and 
<https://journals.plos.org/plosone/s/file?id=ba62/PLOSOne_formatting_sample_title_authors_affiliations.pdf>

A; formatted as requested

2. We note that the grant information you provided in the ‘Funding Information’ and ‘Financial Disclosure’ sections do not match.

When you resubmit, please ensure that you provide the correct grant numbers for the awards you received for your study in the ‘Funding Information’ section.

A: done as suggested

3. We note that you have stated that you will provide repository information for your data at acceptance. Should your manuscript be accepted for publication, we will hold it until you provide the relevant accession numbers or DOIs necessary to access your data. If you wish to make changes to your Data Availability statement, please describe these changes in your cover letter and we will update your Data Availability statement to reflect the information you provide.

A: The data is already deposited in GenBank under the BioProject that we’ve provided as private. We now added the accession number for each genome in the manuscript and they will be immediately released as a publication appears. Therefore, we fully comply with PLOS ONE and NCBI policy for Data Availability.

In the revised version accession number of the genomes (the table below) was placed below Acknowledgements section.

| Strain | BioProject | BioSample | Accession | Study | SRA Accession |
| --- | --- | --- | --- | --- | --- |
| Balcarce_14.2 | PRJNA280853 | SAMN17295281 | CP076243-CP076244 | SRP057321 | SRR14419381,SRR14419382 |
| Vac07.1 | PRJNA280853 | SAMN17295280 | CP076241-CP076242 | SRP057321 | SRR14419379,SRR14419380 |
| 146N4 | PRJNA280853 | SAMN17295279 | CP076237-CP076240 | SRP057321 | SRR14419377,SRR14419378 |
| 9.1_Anguil | PRJNA280853 | SAMN17295278 | CP076235-CP076236 | SRP057321 | SRR14419375,SRR14419376 |
| Balcarce_24.2 | PRJNA280853 | SAMN17295277 | CP076245-CP076247 | SRP057321 | SRR14419387,SRR14419388 |
| 7.1_Anguil | PRJNA280853 | SAMN03470769 | CP076232-CP076234 | SRP057321 | SRR14419385,SRR14419386 |
| Rafaela_II | PRJNA280853 | SAMN03470766 | CP076230-CP076231 | SRP057321 | SRR14419383,SRR14419384 |
| 438/99 | PRJNA280853 | SAMN17295282 | JAHCTZ000000000 | SRP057321 | SRR14419389,SRR14419390 |

4. We note that you have included the phrase “data not shown” in your manuscript.

A: we replaced the first data no shown (line 274 of the revised clean version) by S Table 3, and the second (line 470) was deleted, as text is itself self-explanatory.

5. Please upload a new copy of Figures 1 to 6 as the detail is not clear. Please follow the link for more information: <https://blogs.plos.org/plos/2019/06/looking-good-tips-for-creating-your-plos-figures-graphics/>" <https://blogs.plos.org/plos/2019/06/looking-good-tips-for-creating-your-plos-figures-graphics/>

A; new more defined figures were uploaded

**Reviewers' comments and Reviewer's Responses to Questions**

**Reviewer #1**: This study reports a comparative genomic analysis of eight EHEC strains isolated from cattle in Argentina, that were previously classified into clades based on SNPs. These strains were sequenced using PacBio technology and used adequate bioinformatic software. They made comparative analysis founding virulence genes related to LEE genomic island, toxin effectors and stress resistance. Among novelty information, the authors found prophage distribution between strains which is relatively different to previous reports and were capable to group into families. Its findings are very interesting and give new insights into diversity of this important pathotype.

I only have a few observations and suggestions to make your manuscript better understood:

1. “Pathogenicity island” is a no longer used term, is preferred “genomic Island” even if it harbors virulence genes, my suggestion is to change the terms.

A: corrected as requested

2. When you mentioned “geneious” you are referring to the software so, you should specify it in order to avoid confusion particularly to novel readers into bioinformatics

A: Yes, we are referring to software Geneious. We believed it was clear that was a product because we added the company in brackets, but we added the word “software” to clarify.

3. Shiga-toxin detection and cytotoxicity in Vero cells is a well performed assay and according to previous bibliography and provides important information but it seems to be not related with the title of the manuscript, later in text, the importance of this assay arises so I suggest to justify it when you introduce the results of the experiment

A: We acknowledge the comment of the reviewer and we add this sentence in lines 401-403 (of the revised clean version) “Stx toxin production was analyzed in strains carrying stx2a operon (Fig 5) in order to associate gene content of the encoding prophages with Stx production”

4. All the figures are blurry even if you download it as .tiff format, I´m not sure if they are formatted in the correct specification. It makes difficult to follow the text referring to the figure.

A: The reviewer is right. More defined figures were uploaded for the revised version

5. Lines 229-230. You are talking about cytotoxicity referring to Figure 1, but it is an Artemis map not a toxicity graph

W: we apologize for the mistake. We deleted the sentence about Verocitotoxicity assays and as this information (line 229-230) is redundant with that in Lines 363-368. We prefer to leave it as it is in 363-368 (lines 405-410 in the revised version)

6. Lines 370-375. You are indicating panel (A) and (B) but they are not indicated in the figure, please correct it

A: corrected as indicated

**Reviewer #2**: This manuscript presents a genomic comparison of eight E. coli O157:H7 strains, isolated from the same region of Argentina. Comparative genomic tools were applied to screen for genomic features well-described by this field, including clade typing, prophage analysis, stx2 genotyping, and virulence gene identification. The authors identify characteristics reported before in similar studies, including diverse stx2a prophage but less diversity in stx2c prophage, polymorphisms in stx2-associated genes, and genomic rearrangements. While most of their findings were expected, the experiments were performed properly and for the most part (see below), their interpretations were supported by the data.

Major comments;

1. With hundreds of O157:H7 sequenced genomes available, this seems like a missed opportunity not to perform a larger comparison with other clade 8's. This would add much more value to the paper.

A: We understand that a larger dataset of E. coli genomes is available, however, most of the comparisons made in the present study included some phenotypic information of the analyzed strains or manual comparisons (like alignments) of the data that couldn’t be performed in a dataset of thousands of genomes. We chose to sequence the genomes to completion and use complete closed STEC O157:H7 genomes from NCBI in our analysis because we wanted to compare all aspects of genome diversity including chromosome architecture, complete phage content, plasmids and other mobile genome elements to identify any features that might associate with observed phenotype difference between the Argentinean strains and also those used in the comparison. Such detailed resolution is not available with draft genome sequences, which are the majority on public databases. However, we believe that the present study could be a start point to think how to upgrade comparisons made to include all deposited E. coli genomes and we intend to do this in future studies.

2. In the absence of a deeper genomic analysis, the phrase “sheds light about the genetic basis of hypervirulence” must be removed. This study does not report any experiments that provide new insights into in vivo virulence potential.

A: the sentence was changed to “This genomic analysis may contribute to the understanding of the genetic basis of the hypervirulence of EHEC O157:H7 strains circulating in Argentine cattle”.

3. It appears a decent amount of prophage analysis was done with a “custom Perl script”. Without access to the script, the reader cannot repeat any this portion of the study. This should be made available on GitHub or similar site.

A: The Perl script was only used to parse BLAST comparisons files. But as the reviewer suggested, was uploaded to GitHub repository to facilitate reproduction. It is now available at <https://github.com/arielamadio/Ecoli_parsing_data> and this was also added to the manuscript.

4. What statistical analysis was done to make the “larger prophages” conclusion in lines 261-262?

A: the text about larger prophages associated to unique prophages was moved the end of the Prophage diversity section in lines. We added a text: A tendency was observed that shorter prophages are likely to be associated with higher clustering (**S Fig 3**), as previously described by Shaaban et al [43]. Conversely, larger prophages (>80 kb) were overrepresented among the unique prophages (line 307-309 in the revised clean version).

The association of prophage size with clustering was determined by Spearman Rho test that the association was statistically significant.

5. Line 363: Did you test whether this strain makes Shiga toxin using your ELISA? If not, explain in the paper why.

A: Yes, in ELISA E. coli O157:H7 9.1 Anguil appears as a very low producer while E. coli O157:H7 RafII is a strong producer as shown in our previous paper (Amigo et al, PLoS One. 2015;10. doi:10.1371/journal.pone.0127710, Fig S1).

6. The Discussion is a reiteration of the Results. These two sections should be combined.

W: As indicated by thee reviewer the two sections were combined.

7. Many of the Figures, even when downloaded via the link provided, are really blurry.

W. new more defined figures were uploaded

Minor comments:

1. Lines 34-35: sentence fragment

A: corrected as suggested. Sentence was fragmented to: Clade 8 strains that were classified as hypervirulent. Most of the strains of this clade have a Shiga toxin *stx2a-stx2c* genotype

2. Line 66: stx2h and 2i have been reported as well
A: corrected as suggested to stx_2_ (stx2_a_, stx2_b_, stx2_c_, stx2_d_, stx2_e_, stx2_f_, stx2_g,_ *stx2h and stx2i*)

3. Line 224: temper this language, as there may be additional prophage that weren’t identified by Phaster.

A: Corrected to The number of inserted prophages detected by Phaster, varied from 13 to 16 per genome
