# Supplementary material for: Genomic analysis of shiga toxin-containing Escherichia coli O157:H7 isolated from Argentinean cattle
Source: PLoS One. 2021 Oct 28;16(10):e0258753. doi: 10.1371/journal.pone.0258753 (PMC8553066; doi:10.1371/journal.pone.0258753)
Supplement: Attachment — Submitted filename: Rebuttal letter.docx [file pone.0258753.s009.docx]

|

|

Mark Eppinger
Academic Editor
PLOS ONE

30 September 2021

Ref: PONE-D-21-18023 R1

Genomic analysis of shiga toxin-containing Escherichia coli O157:H7 isolated from Argentinean cattle

Please find attached a revised (R2) version of our manuscript: “Genomic analysis of shiga toxin-containing Escherichia coli O157:H7 isolated from Argentinean cattle”. We thank the reviewers for their comments and suggestions and have modified the manuscript accordingly. Please find below a point-by-point response to each of the concerns raised by the reviewers.

We feel that these changes have significantly enhanced our manuscript, and hope that you will now find it acceptable for publication in *Plos One*.

Yours sincerely

Dr. Angel Cataldi

**Responses to the academic editor:**

Comment/Question (C/Q):"The authors added "stx2h" and "stx2i" as previously suggested, but use an incorrect 2012 reference (these variants were first reported around 2018-2019).  Please see doi:10.1038/s41598-018-25233-x and doi: https:doi.org/10.4315/ 0362-028X.JFP-18-291."

Answer (A): the two reference were added in line 67 as [8] and [9].

C7Q: Also, a more recent article reports on stx2k (Yang, X. et (2020). Escherichia coli strains producing a novel Shiga toxin 2 subtype circulate in China. Int J Med Microbiol 310, 151377.).

A: The reference was added as [10] in line 67

. Review Comments to the Author

C7Q: Reviewer #1: As mentioned in past review, this study worth to be communicated. All recommendations have been addressed

A: questions of Reviewer 2 have been addressed under Responses to the academic editor:
